# Development of the CORE-Kids core set of outcome domains for studies of childhood limb fractures

**DOI:** 10.1302/0301-620X.103B.BJJ-2020-2321.R2

**Published:** 2021-12-01

**Authors:** Ben A. Marson, Joseph C. Manning, Marilyn James, Simon Craxford, Sandeep R. Deshmukh, Daniel C. Perry, Benjamin J. Ollivere, The CORE-Kids collaborative group

**Affiliations:** 1 Department of Trauma and Orthopaedics, University of Nottingham, Nottingham, UK; 2 Faculty of Medicine & Health Sciences, University of Nottingham, Nottingham, UK; 3 Institute of Translational Medicine, University of Liverpool, Liverpool, UK

**Keywords:** Core outcome set, Fracture, Outcome, Bone, Upper limb, Lower limb, limb fractures, Lower extremity, clinical outcomes, upper limb fractures, deformity, lower limb fractures, Paediatric Orthopaedic, Upper extremity, Orthopaedic Surgeons, paediatric orthopaedic surgeons

## Abstract

**Aims:**

The aim of this study is to develop a core set of outcome domains that should be considered and reported in all future trials of childhood limb fractures.

**Methods:**

A four-phase study was conducted to agree a set of core outcome domains. Identification of candidate outcome domains were identified through systematic review of trials, and outcome domains relevant to families were identified through semi-structured interviews with 20 families (parent-child pairing or group). Outcome domains were prioritized using an international three-round Delphi survey with 205 panellists and then condensed into a core outcome set through a consensus workshop with 30 stakeholders.

**Results:**

The systematic review and interviews identified 85 outcome domains as relevant to professionals or families. The Delphi survey prioritized 30 upper and 29 lower limb outcome domains at first round, an additional 17 upper and 18 lower limb outcomes at second round, and four additional outcomes for upper and lower limb at the third round as important domains. At the consensus workshop, the core outcome domains were agreed as: 1) pain and discomfort; 2) return to physical and recreational activities; 3) emotional and psychosocial wellbeing; 4) complications from the injury and treatment; 5) rturn to baseline activities daily living; 6) participation in learning; 7) appearance and deformity; and 8) time to union. In addition, 9a) recovery of mobility and 9b) recovery of manual dexterity was recommended as a core outcome for lower and upper limb fractures, respectively.

**Conclusion:**

This set of core outcome domains is recommended as a minimum set of outcomes to be reported in all trials. It is not an exhaustive set and further work is required to identify what outcome tools should be used to measure each of these outcomes. Adoption of this outcome set will improve the consistency of research for these children that can be combined for more meaningful meta-analyses and policy development.

Cite this article: *Bone Joint J* 2021;103-B(12):1821–1830.

## Introduction

There is a growing awareness regarding the need for careful outcome selection in clinical research. Research is expensive and time-consuming, and may be wasteful if the findings from different studies are not comparable.^1,2^ Standardized outcomes, measured in all research studies relating to a specific condition, offer a means to reduce this research waste and ensure that studies report outcomes that can be combined in meaningful meta-analyses.^3,4^

The objective of designing a core set of outcome domains is to agree the outcome domains that need to be measured in every research study. These are the broad “what to measure” constructs, such as pain or function. The Core Outcome Measures in Effectiveness Trials (COMET) Initiative has developed guidelines and methodology for the generation of these standardized sets of outcomes in the form of core outcome sets.^5-7^ In a core outcome set, an agreed collection of outcomes is identified as the minimum reporting standard for every trial. Additional outcomes may be measured alongside this core set by researchers to capture condition-specific characteristics, but all trials in a field should report the minimum set to facilitate meta-analysis and prevent reporting bias.

Once the core set of outcome domains is agreed, the COMET Initiative and COnsensus-based Standards for the selection of health Measurement INstruments (COSMIN) have published additional guidelines relating to the selection of outcome tools (such as patient-reported outcomes) to measure these domains.^5^ The prerequisites for recommending outcome tools are an agreed set of core outcome domains and validation studies for the candidate tools.

There is a need for a core outcome set for childhood fractures, as currently there is little consistency in the reporting of clinical outcomes.^8-11^ This propagates research waste as study outcomes cannot be combined into pooled meta-analyses or reliable clinical guidelines. The first part of achieving a core outcome set is to agree the core set of outcome domains that need to be measured. The aim of this study was to establish this core set of outcome domains that should be considered in research involving school-aged children with limb fractures.

## Methods

### Scope and design

The study protocol was registered on the COMET database and the protocol was prospectively published.^12,13^ Study reporting is consistent with the COS-STAR guidelines (Supplementary Material).^14^ As stated in the study protocol,^15^ the scope of this core outcome set was set by the steering group in collaboration with the National Institute for Health Research (NIHR) trauma trials network as: Setting: research studies; Health condition: fractures to the appendicular skeleton (i.e. limbs, pelvis, and shoulder girdle but not spine, ribs, or head) excluding children with multiple injuries. The core outcome set will be divided into three modules: a central ‘all fractures’ set, an ‘upper limb’ set, and a ‘lower limb’ set. The target population is school-aged children (aged 5 to 16 years). It is anticipated that infants (0 to 4 years) and older adolescents (17 to 18 years) may share common core outcomes, but this cannot be assumed and should be confirmed in future work. Target interventions are treatment for fractures, both involving surgical and non-surgical (conservative) techniques.

The study was formed of three principal components to develop consensus on what outcome domains should be measured. These were performed sequentially and addressed: what are the outcome domains that may be relevant for children with limb fractures?; what are the most important of these outcomes?; and which outcomes should be included in the core outcome set?

Ethical approval was awarded on 06/08/2019 by the North London—Hampstead REC (HRA/REC IRAS number 262503).

### Identification of candidate outcome domains

Candidate outcome domains that may be relevant for children with limb fractures were identified through a systematic review of childhood fracture trials and through an interview study with parents and children. As this phase of the study was exploratory to gather a broad list of candidate outcome domains for the subsequent phases of the study, the scope of the systematic review and interviews was broadened to include children of all ages (0 to 18 years).

The systematic review was performed with the searches completed on the 8 August 2019 to identify all outcomes reported in randomized trials of childhood fractures. A search was performed of OVID Medline, EMBASE, and Cochrane CENTRAL. The detailed methods, search terms, and results have been published elsewhere.^16^ All outcomes and patient-reported outcome measures reported were mapped onto the World Health Organization International Classification of Functioning, Disability and Health (WHO ICF) and subsequently formed the basis for the Delphi survey.^17^

The systematic review identified 100 eligible trials, for which outcome domains were extracted.^16^ From these, 525 different descriptions of outcome domains were extracted which were mapped onto 52 outcome domains in the WHO ICF framework. The outcome domains that were reported in more than 40% of upper limb trials were sensation of pain, mobility of joint functions (range of motion (ROM)), and the structure of upper limb (radiograph appearance). The outcome domains that were reported in more than 40% of lower limb trials were sensation of pain, mobility of joint functions (ROM), structure of lower limb (radiograph appearance) of the leg, and healthcare cost.

Additional relevant outcome domains for children with limb fractures were elicited through a series of qualitative semi-structured interviews. These were undertaken with a parent and child together, where the child had fractured a bone in an arm or leg. Detailed methods have been published elsewhere.^15^ In brief, children with fractures were sampled from outpatient clinics with purposeful sampling to generate a diverse group of ages, fracture types, and experience of different treatments. Interviews were conducted and recorded by researchers trained in qualitative methods and analyzed using a content analysis. All activities and outcomes identified by parents and children were extracted by two researchers and mapped onto the WHO ICF framework using established linking rules.^18-20^ Sampling continued until data saturation was reached as defined by two consecutive interviews that yielded no additional WHO ICF domains. Key outcome domains were identified as those reported in at least half of the interviews.

A total of 20 interviews were conducted with each parent-child pairing or group. The demographic details of the participants are shown in Supplementary Table i. There were 14 children with current upper limb fractures and six with current lower limb fractures, equally balanced between males and females; 13 children were aged three to ten years and seven children were aged 11 to 16 years. In the sample, 15 children were treated with cast immobilization (with or without surgery), and five treated in a sling or removable splint. Seven children were admitted to hospital for the treatment of the fracture. Children were accompanied by their mother in 17 interviews, by their father in one interview, and by both parents in two interviews.

### Prioritization of outcome domains

The outcome domains from the systematic review and qualitative interviews were pooled to generate a comprehensive list of outcomes to be evaluated using an online Delphi consensus survey. The Delphi survey was piloted with item reduction of duplicate outcome domains or domains focusing on environmental support. Explanatory statements were added where necessary which were selected in conjunction with a public and professional participation panel of two parents and two doctors. This left a list of 68 outcome domains that were evaluated for both upper and lower limb fractures.

The Delphi survey consisted of three rounds, each lasting six to eight weeks. Each round aimed to identify consensus for the importance of each outcome domain for children with upper or lower limb fractures. Three rounds were selected to improve the chance of developing consensus while minimizing attrition from survey fatigue.^21^ A wide range of international stakeholders contributed to the Delphi study panel of 205 participants, including parents, doctors, therapists, teachers, and researchers, to cover a breath of experience surrounding the care of children with limb fractures. The professional and geographical distribution of panellists is shown in Supplementary Tables iii and iv. Participants were approached using local networks and through national societies including the British Society of Children’s Orthopaedic Surgeons, the Paediatric Orthopaedic Society of North America, the Paediatric Orthopaedic Society of India, Paediatric Orthopaedic Practitioners Society, Paediatric Orthopaedic Society of New Zealand, Nederlandse Orthopaedische Vereniging, and Health Care Play Specialist Education Trust. The Delphi Study was completed using an online survey using the onlinesurveys.ac.uk interface (JISC, UK)

For each outcome, panellists were invited to assign scores from 1 (not important) to 9 (very important). Each outcome domain was scored twice: once for upper limb fractures and once for lower limb fractures.

To minimize attrition, outcomes that reached consensus in or out were removed between rounds. The pre-specified consensus thresholds were met for ‘consensus in’ if > 70% panellists assigned an outcome with a score of 7 to 9 and ≤ 15% of panellists scored the outcome 1 to 3, and ‘consensus out’ if > 70% panellists assigned an outcome with a score of 1 to 3 and ≤ 15% of panellists scored the outcome 7 to 9. This threshold was set to be consistent with other core outcome set thresholds and protects the divergent views of minority stakeholders from being overridden by a larger stakeholder group.^22-24^

Feedback was provided between the first and second round as multiple-combined feedback from all groups. Following stakeholder feedback during the second round, feedback was provided between the second and third round, with parent scores and professional scores presented as separated bar graphs.^25,26^

A free-text question in the first round of the Delphi survey permitted panellists to recommend any additional outcomes for inclusion in later rounds of the survey.

### Which outcomes should be included in the core outcome set?

The results from all phases of this study were compiled and taken to a final consensus meeting hosted at the University of Nottingham in March 2020. This meeting comprised representatives from all key stakeholder groups and included 13 surgeons, two advanced paediatric nurses, four therapists, two researchers, and nine parents or young people from the UK with experience of limb fractures.

Through additional work with our patient, parent, and public advisory group, we sought to ensure the consensus group methodology was widely accessible to families. The consensus meeting structure and voting system was amended from the original protocol to adopt the principles of the Technology of Participation consensus workshop method.^27^ The Technology of Participation consensus workshop was developed by the Institute of Cultural Affairs (ICA) in the 1970s as a method of empowering communities to develop local action plans to address local development issues around the world.^28,29^ The methods have been used in international development,^28-30^ healthcare,^31^ and by the United Nations^32^ to facilitate meaningful conversations and empower diverse stakeholders around common goals.

The meeting was divided into five stages and facilitated by a researcher who did not participate in the voting. The context of the meeting was set through an oral presentation of the research question, and written participant books. The books contained details of the outcome domains which were relevant according to the systematic review and interviews, and which had been prioritized in the Delphi study.

Using the outcomes from the Delphi survey, participants were asked individually to identify key outcome domains for inclusion in the core set of outcome domains. Participants were not constrained by outcome domains featured in the previous studies but were encouraged to use the findings to contextualize their own experience and views. These were prioritized through facilitated discussion within four groups of six to eight participants. The sample size was selected to balance the need for a manageable number of key domains for discussion while maximizing the diversity of experience available within the groups. The use of small groups was selected to minimize the impact of any dominant individuals within the consensus group.

The key outcome domains were clarified and then sequentially clustered with input from the whole group. During this step, outcome domains could be clustered by duplication and by meaning by group agreement. Once all the key outcome domains had been presented and clustered, each cluster was assigned an abstract symbol. Each symbol was identified and named by the group as a descriptive domain that encompassed the features of the clustered concepts. Each named cluster was considered a potentially relevant outcome domain for the core outcome set.

A further visual prioritization step was performed with participants able to distribute seven points to the outcome domains that they were most keen to include in the core set. Following this visual feedback, the core set of outcome domains was agreed using anonymous electronic voting. During the final voting, participants could vote each outcome domain for inclusion in an upper limb or lower limb core set. A pre-specified threshold of > 70% was set for outcomes to be included in the core set.^15^ A sensitivity analysis was performed to identify the effect of the majority stakeholder group by first removing the scores of surgeons and then exclusively including scores of partners and patients.

## Results

### Identification of candidate outcome domains

**Fig. 1:**
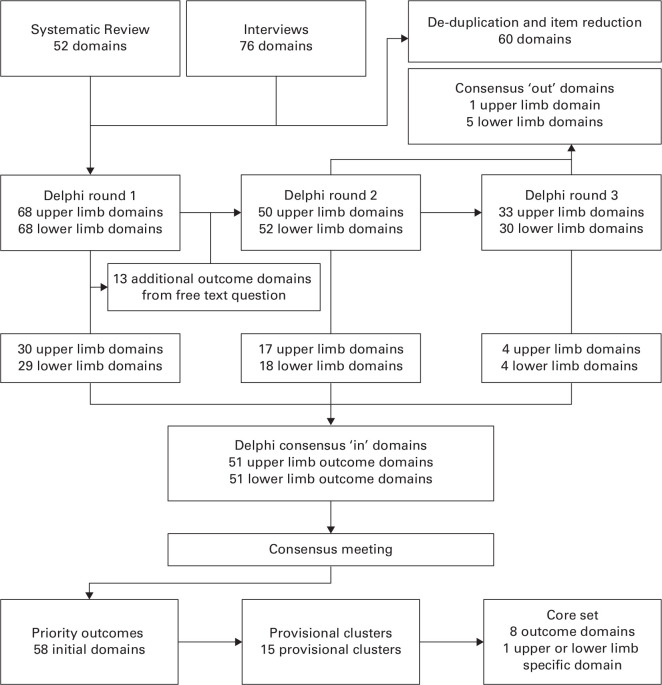
Flowchart of outcome domains considered for inclusion in the core set of outcome domains.

A flowchart demonstrating the study progress is shown in Figure 1. Analysis was performed on 20 parent-child interviews. One transcript was unavailable due to a recording error, but analysis was performed on the field notes. The mean length of recordings was 13.3 minutes (standard deviation (SD) 5). Extraction of verbatim outcomes from transcripts yielded 202 outcome domains from children and 575 outcome domains from parents. Following linkage to the WHO ICF framework, 11 body function, six body structure, 21 activity and participation, and seven environmental outcomes were identified by parents and children as relevant to them, in addition to the 52 outcomes identified in the systematic review of trials (Table I).

**Table I.** Additional outcome domains that had not been identified during systematic review, but were identified by parents and children during interviews.

ICF outcome domain	Upper limb (n = 14)	Lower limb (n = 6)
**Body function**		
Consciousness functions	0	3
Temperament and personality function (extraversion)	1	1
Temperament and personality function (agreeableness)	1	1
Temperament and personality function (confidence)	1	0
Sleep functions (maintenance of sleep)	0	1
Proprioceptive function	0	3
Touch function	0	1
Blood vessel function	0	3
Respiratory function	0	3
Exercise tolerance functions	0	1
Muscle tone functions	0	2
**Activities and Participation**		
Handling stress	0	1
Producing drawings and photographs	0	3
Sitting	2	0
Standing	2	0
Transferring oneself	3	5
Using communication devices and techniques	0	3
Manipulating	0	3
Climbing	4	2
Running	3	2
Swimming	2	5
Moving around using equipment (wheelchair/crutches)	3	0
Driving human-powered transportation	1	1
Caring for teeth	0	4
Caring for hair	0	2
Putting on footwear	2	3
Shopping	2	1
Relating with persons in authority	1	0
Informal relationships with friends	2	4
Recreation and leisure: play	2	7
Recreation and leisure: sport	4	8
Recreation and leisure: arts and culture	1	4
**Environmental factors**		
Food	1	3
Assistive products and technology for personal use in daily living	1	3
Immediate family	3	7
Products and technology for personal indoor and outdoor mobility and transportation	3	1
Friends	1	2
Health professionals	4	3
Health services, systems, and policies	1	6
**Body structure**		
Structure of the nervous system, other specified	0	1
Structure of cardiovascular system	0	4
Structure of respiratory system	0	3
Structure of head and neck	1	3
Structure of vertebral column	1	0
Structure of areas of skin	1	1

ICF, International Classification of Functioning, Disability and Health.

There were eight common outcome domains identified in over half the interviews: sensation of pain (19 interviews), emotion functions (15 interviews), school education (13 interviews), recreation and leisure: sport (12 interviews), washing oneself (11 interviews), sleep functions (ten interviews), eating (ten interviews), and support from immediate family (ten interviews).

For the 14 upper limb fracture interviews, the additional outcome domains that were identified in seven or more interviews were structure of upper limb (nine interviews), dressing (eight interviews), and recreation and leisure: play (seven interviews). Eating was reported in six upper limb fracture interviews.

During the six lower limb fracture interviews, nine additional common outcome domains were identified in three or more interviews. These were walking (five interviews), toileting (five interviews), climbing (four interviews), support from health professionals (four interviews), structure of lower limb (four interviews), transferring oneself (three interviews), running (three interviews), moving around using equipment (three interviews), and support from products and technology for personal indoor and outdoor mobility and transportation (three interviews).

### What are the most important of these outcomes?

Following the first-round Delphi survey, 30 upper limb and 29 lower limb outcome domains met the consensus threshold for inclusion. In total, 13 additional outcome domains were added to the second-round Delphi survey following analysis of free-text responses. A further 17 upper limb and 18 lower limb outcomes met the consensus threshold after the second-round survey. A further four upper limb and four lower limb outcomes met the consensus threshold after the third-round study. Scores for all ‘consensus-in’ outcomes are shown in Table II and Table III, with ranges of scores displayed in box and whisker plots in the Supplementary Material.

**Table II.** Upper limb outcome domains identified as consensus in during Delphi survey.

Outcome	Round outcome accepted	% score 7 to 9 in round 1	% score 7 to 9 in round 2	% score 7 to 9 in round 3
General hand and arm use	1	96	–	–
Writing	1	96	–	–
Fracture union or healing	1	95	–	–
Return to play	1	95	–	–
Range of motion of the limb	1	94	–	–
Pain	1	94	–	–
Lifting and carrying objects	1	94	–	–
Return to sport	1	91	–	–
Ability to perform fine hand tasks	1	90	–	–
Return to creative activities	1	89	–	–
The ability of a child’s blood vessels to maintain normal function	1	89	–	–
Completion of general daily tasks	1	89	–	–
Caring for body parts	1	88	–	–
Ability to participate in school education	1	88	–	–
Dressing	1	87	–	–
Sense of touch	1	86	–	–
Ability to use the toilet	1	85	–	–
The stability of the joint that is next to the broken bone	1	83	–	–
Washing	1	83	–	–
Parental satisfaction	1	82	–	–
Sensations of pins and needles in the arm or leg	1	80	–	–
Drinking	1	79	–	–
Muscle power or strength	1	78	–	–
Ability to put on shoes and socks	1	78	–	–
Cosmetic deformity	1	78	–	–
Growth	1	77	–	–
Carrying out daily routine	1	75	–	–
Eating	1	75	–	–
Radiograph appearance of limb	1	75	–	–
Ability to transfer	1	71	–	–
Complications of surgery or treatments*	2	–	96	–
Return to normal physical function*	2	–	95	–
Quality of life for the child*	2	–	95	–
Refracture or re-injury*	2	–	88	–
Ability to throw (e.g. a ball)*	2	–	86	–
Child satisfaction*	2	–	86	–
Long-term complications (e.g. arthritis as an adult)*	2	–	85	–
Death	2	70	85	–
Emotional distress for the child	2	–	82	–
Ability to draw	2	66	82	–
Proprioceptive function - the child’s ability to know the position of their arm or leg	2	64	80	–
Cost to the family	2	70	79	–
Delay from time of injury to receiving definitive treatment*	2	–	76	–
Number of days off work for parents	2	69	76	–
Length of stay in hospital*	2	–	75	–
Ability to swim	2	57	72	–
Duration of immobilization (time in a cast/pop/splint)	2	63	71	–
Swelling of the limb	3	67	59	79
The need to use equipment to move around (e.g. wheelchair or crutches)	3	66	67	74
Sleep	3	56	63	74
Radiation dose*	3	–	55	71

* Outcomes added to the round 2 survey following analysis of free-text responses in round 1.

**Table III.** Lower limb outcome domains identified as consensus in during Delphi survey.

Outcome	Round outcome accepted	% score 7 to 9 in round 1	% score 7 to 9 in round 2	% score 7 to 9 in round 3
Walking	1	98	–	–
Pain	1	96	–	–
Fracture union or healing	1	96	–	–
Return to play	1	95	–	–
Return to sport	1	94	–	–
Walking gait or pattern	1	92	–	–
Growth	1	90	–	–
Range of motion of the limb	1	90	–	–
Changing body position (e.g. going from sitting to standing)	1	90	–	–
The ability of a child’s blood vessels to maintain normal function	1	88	–	–
Ability to run	1	88	–	–
Ability to move around (generally)	1	87	–	–
Ability to participate in school education	1	86	–	–
Moving around in different locations (e.g. within the house or outdoors down a street)	1	86	–	–
The stability of the joint that is involved in or next to the broken bone	1	85	–	–
Completion of general daily tasks	1	85	–	–
The need to use equipment to move around (e.g. wheelchair or crutches)	1	85	–	–
Muscle power or strength	1	83	–	–
Parental satisfaction	1	83	–	–
Ability to transfer (e.g. from a bed into a chair)	1	82	–	–
Ability to use the toilet	1	81	–	–
Radiograph appearance of limb (e.g. angulation)	1	78	–	–
Exercise tolerance	1	77	–	–
Cosmetic deformity (appearance of the limb)	1	76	–	–
Sensations of pins and needles in the arm or leg	1	76	–	–
Sense of touch	1	74	–	–
Number of days off work for parents	1	72	–	–
Ability to put on shoes and socks	1	72	–	–
Cost to the family	1	71	–	–
Return to normal physical function*	2	–	96	–
Complications of surgery or treatments*	2	–	96	–
Quality of life for the child*	2	–	96	–
Ability to jump*	2	–	90	–
Long-term complications (e.g. arthritis as an adult)*	2	–	90	–
Refracture or re-injury*	2	–	88	–
Child satisfaction*	2	–	86	–
Death	2	71	85	–
Emotional distress for the child*	2	–	83	–
Proprioceptive function - the child’s ability to know the position of their arm or leg	2	67	79	–
Delay from time of injury to receiving definitive treatment*	2	–	78	–
Duration of immobilization (time in a cast/pop/splint)	2	68	76	–
Riding a bike	2	59	75	–
Length of stay in hospital*	2	–	75	–
Ability to swim	2	58	73	–
Cost to the hospital	2	61	73	–
Carrying out daily routine (able to plan and complete separate actives through the day)	2	69	72	–
Ability to climb	2	58	71	–
Swelling of the limb	3	69	58	81
Sleep	3	56	64	76
Radiation dose*	3	–	57	72
Happiness of the child*	3	–	62	71

* Outcomes added to the round 2 survey following analysis of free-text responses in round 1.

### Which outcomes should be included in the core outcome set?

Due to travel restrictions related to the COVID-19 outbreak, four paediatric orthopaedic surgeons had to attend electronically via Skype for Business (Microsoft, USA) with a shared electronic version of the sticky wall.

A detailed breakdown of the consensus workshop steps and outputs is available in the Supplementary Material. To develop a core set of outcome domains, participants evaluated the 51 upper and lower outcome domains that reached the Delphi consensus threshold and used these to develop a list of initial outcome domains for inclusion in the core outcome set. During the facilitated small group discussion an initial 58 outcome domains were proposed for the core set of outcome domains.

In the whole group discussion, the 58 proposed outcome domains were clustered into 15 provisional clusters. Each group of outcome domains was then evaluated and named through group consensus to form the list of named clusters.

**Fig. 2:**
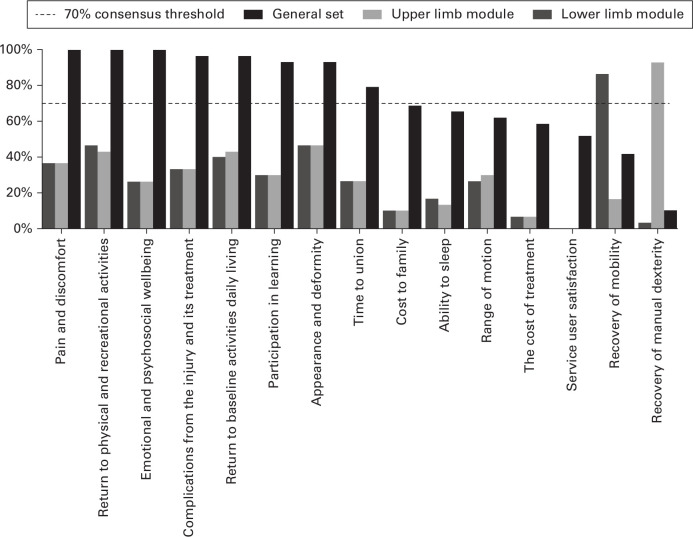
Voting outcomes from consensus workshop identifying the general core outcome set for children’s fractures and the upper and lower limb modules, ordered left to right by percentage voting for the score to be included in the general set of core outcome domains.

From the list of named clusters, the core set was agreed through visual prioritization and an electronic vote of participants. Scores for each outcome domain are demonstrated in Figure 2. The following outcomes met the consensus threshold and were recommended by the consensus group for inclusion in the general core outcome set: pain and discomfort (100% (95% confidence interval (CI) 88.4% to 100%)); return to physical and recreational activities (100% (95% CI 88.4% to 100%)); emotional and psychosocial wellbeing (100% (95% CI 88.4% to 100%)); complications from the injury and its treatment (96.7% (95% CI 82.8% to 99.9%)); return to baseline activities of daily living (96.7% (95% CI 82.8% to 99.9%)); participation in learning (93.3% (95% CI 77.9% to 99.2%)); appearance and deformity (93.3% (95% CI 77.9% to 99.2%); and time to union (80.0% (95% CI 61.4% to 92.3%)).

Body area-specific outcomes were: recovery of mobility (lower limb fractures 100% (95% CI 88.4% to 100%)) and recovery of manual dexterity (upper limb fractures 100% (95% CI 88.4% to 100%)).

The core set of outcome domains was shown to be robust in sensitivity analysis (Supplementary Material) with all included outcomes exceeding the 70% consensus thresholds when analyzed without doctors and when patients and parents were analyzed separately. In the sensitivity analysis, cost to family, ability to sleep, and range of motion were identified as important to non-doctor participants and to parents and patients having exceeded the 70% consensus threshold in these groups.

## Discussion

The use of core outcome sets has been shown to improve the standardization of reporting in other medical specialities and can therefore reduce reporting bias in studies and improve the ability of systematic reviews and policymakers to synthesize trials.^2,33,34^ This study has delivered a core set of outcome domains for use in childhood fractures with eight core outcome domains for all fractures, and one additional outcome domain relevant for each body region (i.e. upper and lower limb fractures). Participants were not asked to order these outcome domains or assign relative priority to any domain, so all outcome domains that met the consensus threshold of 70% should be regarded as equally important.

This study has been performed according to the international guidance from the COMET Initiative, with the sequential objective to identify, review, and decide on the important outcomes.^6,35^ At each step, new outcome domains were identified that were relevant to different stakeholder groups, highlighting the need for the distinct components of this study and the need for involvement of multiple stakeholders. This echoes the findings of Harman et al,^36^ who found that early engagement with patients with otitis media was valuable in maintaining the views of this critical stakeholder group.

This study was designed to mitigate some of the potential problems with the generalizability of a core outcome domain set. The systematic review and interviews were complementary in producing a broad list of candidate outcomes for inclusion in the Delphi study. The interviews were limited by the inclusion of families from a single centre in the UK with the majority of patients having white British ethnicity, nonoperative management, upper limb injuries, and mostly mothers in the sample. For this reason, the interviews are not generalizable in isolation but added complementary information to the systematic review to provide candidate outcome domains for consideration in the Delphi list. In particular, the low numbers of children with experience of lower limb injuries and fractures treated with surgical management means that this component of the study is not generalizable to the whole population of children with fractures. Caution must be applied in extrapolating the frequencies of responses to all children with fractures. Mitigation for this was performed by including the further consensus steps, and by viewing all item domains from the interviews as potentially relevant rather than prioritizing by frequency of responses, which would be at risk of bias due to sample size and selection. The potential for the systematic review and interviews to miss potentially important outcome domains relevant in different settings was also addressed with the inclusion of the free-text question in the first Delphi round, to capture any additional outcomes relevant to members of the Delphi panel.

The use of the Technology of Participation method for group consensus is used less commonly than other consensus methods such as the iterative method or nominative group technique. The method allowed all participants to contribute, formulate, and agree a set of core outcome domains, and allowed minority stakeholders to contribute equally with a formal structure to the consensus development and anonymous voting. There is the potential for a bias to be introduced by having a majority stakeholder group of clinicians, which has been investigated in a sensitivity analysis of voting by comparison of different groups and calculation of binomial exact confidence intervals (Supplementary Material).

The systematic review, interviews, and Delphi study led to a long list of outcome domains that were considered important. However, it would be unreasonable for clinical studies to collect data on all these outcome domains, hence the need for the consolidated core outcome set. Additional outcomes, beyond the core outcome set, may also become more relevant for specific fracture types or specific patient groups. The core outcome set is therefore a minimum set of outcomes that should be reported in all robust prospective clinical research to improve reporting consistency and comparability. Authors of future studies should be encouraged to report all these outcome domains or consider justifying why these core outcome domains have not been reported.

This core set has identified the minimum outcome domains that should be measured in all future trials relating to childhood limb fractures. The outcome domains that have been recommended in the core set have been clustered and named by the consensus group, although further work is required to establish the optimal technique to measure each domain. These domains can be used immediately in the design of studies to ensure comparability and relevance of study outcomes and may be measured by selecting appropriate outcome tools. In a recent systematic review of validation studies, insufficient evidence was found to recommend any specific outcome tools to measure physical function or quality of life.^37^ Future studies should report all domains identified within this set as part of their outcome assessment using outcome tools appropriate to the population and specific fracture being studied until this validation is completed and agreement can be reached.

In the development of this core set of outcome domains, attempts were made to develop international applicability, particularly to middle- and low-income countries which have been under-represented in previous core outcome set studies.^38^ In the Delphi panel, six of the 23 represented countries were low- or middle-income as defined by the World Bank. For logistical reasons, the interviews and consensus meeting were held in the UK but were informed by the systematic review that included studies from 27 different countries and a Delphi panel with representatives from 23 countries. Unfortunately, due to travel restrictions the consensus group could not be fully international.

Attrition during Delphi surveys is a recognized issue in this methodology, particularly with Delphi surveys with multiple items for panellists to rate.^39,40^ The overall retention of 69.8% between first and third round of this study is reasonable and consistent with similar studies.^41-43^ This may be a source of bias, particularly where stakeholder groups have different attrition rates.^44^ We accept that the differential attritional rates between our different stakeholders may have had an effect on the included outcomes in the Delphi study (shown in Supplementary Material). We attempted to reduce attrition by using repeated reminders. The impact of any attritional related bias was minimized with the inclusion of the face-to-face consensus meeting, where the Delphi results were used as the basis for discussions.

The primary aim of this study was to deliver a set of core outcome set for use in robust prospective trials involving school-aged children (aged 5 to 15 years) with limb fractures. This core outcome set may also be a useful guide for data collection in clinical practice, as a guide to important outcomes to monitor following injury, and could be embedded in injury registries. We are unable to generalize the results to children aged younger than five years, older than 15 years, or in the setting of polytrauma.

In conclusion, we have developed a core outcome set for use in research trials of limb fractures in school-aged children. Pain and discomfort, return to physical and recreational activities, emotional and psychosocial wellbeing, complications from the injury and its treatment, return to baseline activities daily living, participation in learning, appearance and deformity, and time to union should be measured and reported in all future research studies involving children with these injuries, to improve the consistency between studies and minimizing reporting bias.

## Endorsement

This core set of outcome domains has been endorsed by the British Society of Children’s Orthopaedic Surgeons, The Orthopaedic Trauma Society, and the Nederlandse Orthopaedische Vereniging as the reporting standard for clinical studies of childhood limb fractures.

**Take home message**

- This paper presents a core set of outcome domains to be measured in all future studies of childhood limb fractures.

- Further work is required to evaluate the measurement tools to capture these outcomes.
